# Erosive Arthritis, Fibromatosis, and Keloids: A Rare Dermatoarthropathy

**DOI:** 10.1155/2018/3893846

**Published:** 2018-04-22

**Authors:** Fawad Aslam, Jonathan A. Flug, Yousif Yonan, Shelley S. Noland

**Affiliations:** ^1^Division of Rheumatology, Department of Internal Medicine, Mayo Clinic, Scottsdale, AZ, USA; ^2^Department of Radiology, Mayo Clinic, Scottsdale, AZ, USA; ^3^Department of Dermatology, Mayo Clinic, Scottsdale, AZ, USA; ^4^Division of Plastic Surgery, Department of Surgery, Mayo Clinic Hospital, Phoenix, AZ, USA; ^5^Department of Orthopedic Surgery, Mayo Clinic Hospital, Phoenix, AZ, USA

## Abstract

Polyfibromatosis is a rare disease characterized by fibrosis manifesting in different locations. It is commonly characterized by palmar fibromatosis (Dupuytren's contracture) in variable combinations with plantar fibromatosis (Ledderhose's disease), penile fibromatosis (Peyronie's disease), knuckle pads, and keloids. There are only three reported cases of polyfibromatosis and keloids with erosive arthritis. We report one such case and review the existing literature on this rare syndrome.

## 1. Introduction

Polyfibromatosis is a rare disease characterized by fibrosis manifesting in different locations. It is commonly characterized by palmar fibromatosis (Dupuytren's contracture) in variable combinations with plantar fibromatosis (Ledderhose's disease), penile fibromatosis (Peyronie's disease), knuckle pads, and keloids [1]. The aforementioned are all categories of superficial fibromatoses. Coexistence of fibromatosis and keloid formation is also very rare [2, 3], and it is not entirely clear if keloids are formally part of the polyfibromatosis syndrome. Nevertheless, this association is rare. To identify previous reports, a nonrestricted PubMed search was carried out using the key words Dupuytren's contracture, keloid, fibrosis, arthritis, osteolysis, erosive arthritis, and their varying combinations. Bibliographies of identified relevant studies were also reviewed for further pertinent cases. A few case reports have described presence of erosive and/or osteolytic disease in patients with polyfibromatosis [4–6]. There are only three reported cases of simultaneous occurrence of fibromatosis, keloids, and erosive arthritis, all in males [7–9]. Interestingly, the keloid formation is also spontaneous in these cases. We report a fourth such case and review the existing literature.

## 2. Case Presentation

A 23-year-old male presented to the primary care clinic to establish care after relocating to Arizona. His past medical history was significant for severe, mostly spontaneous, keloid formation since puberty. He complained of a right ring finger mass and a left ring finger deformity, bilateral foot pain, and worsening keloids and skin nodules.

Over the last four months, he had noticed a spontaneous, slowly enlarging mass on his right ring finger. It bothered him when directly pressed upon or during rock climbing. There were no associated neurological symptoms, redness, or erythema. He also reported a contracture in the left ring finger over the last six months. He had undergone a left small finger proximal interphalangeal joint arthrodesis for presumed camptodactyly at age 16. This surgery was complicated by fibromatosis and keloid scar formation, ultimately leading to amputation of the small finger. He now complained of excessive scar formation at that amputation site.

He gave a two-year history of bilateral forefoot pain. His right fourth toe was the most painful. He reported that this toe had become very stiff over this time. Other toes were involved as well, and prolonged standing exacerbated the symptoms. He did not report any redness, warmth, or swelling. He denied any acute joint pain episodes and hand or back pain.

With respect to his keloids and skin nodules, he had had them since puberty. He noted that some were related to sites of minor trauma, and others were spontaneous. He had multiple large keloids involving the chest, back, arms, and legs. Recently, some had involved the toes as well. He denied any genital involvement. Past treatments included intralesional steroid injections and radiation therapy with temporary improvements.

He was a lifelong nonsmoker and drank alcohol socially. He was an avid rock-climber. His family history was significant for keloid formation in his grandfather. A maternal cousin had rheumatoid arthritis. On review of systems, he denied any fatigue, morning stiffness, night sweats, weight loss, inflammatory eye disease, cough, shortness of breath, back pain, nephrolithiasis, Raynaud's phenomenon, or blood dyscrasias. He did not take any chronic medications.

**Figure 1:**
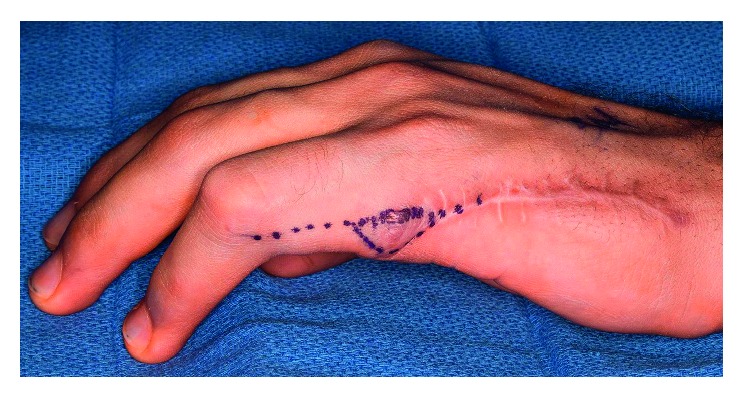
Severe left ring finger contracture with palmar fibromatosis and keloid scar formation.

**Figure 2:**
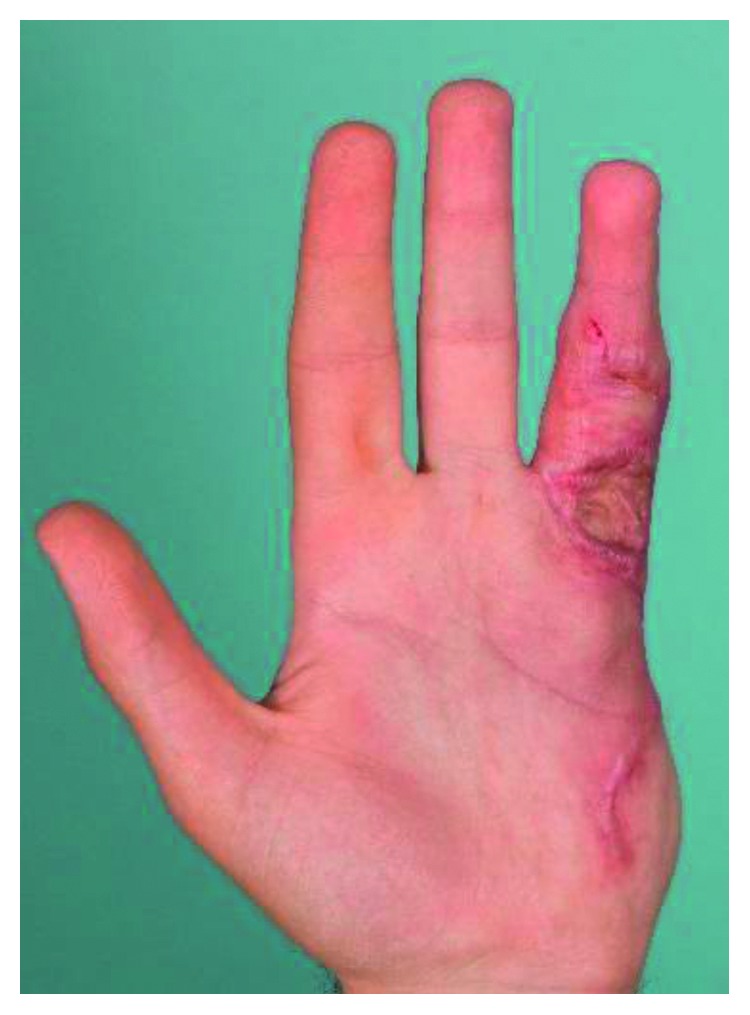
Severe left ring finger contracture with palmar fibromatosis and keloid scar formation.

**Figure 3:**
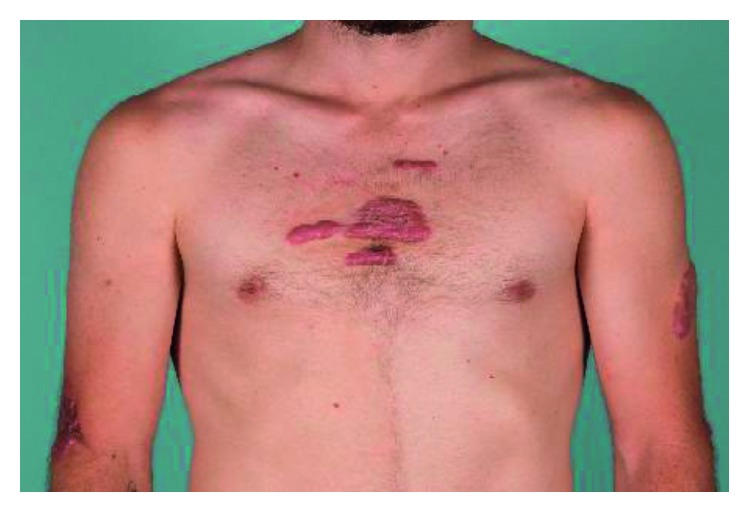
Keloids and hypertrophic scars on the chest and arms.

On examination, he was afebrile with normal vital signs and appropriate weight. There was no glandular swelling or gingival hypertrophy. Hand examination showed severe left ring finger proximal interphalangeal (PIP) joint contracture of 80°, with palmar fibromatosis and keloid scar formation (Figures 1 and 2). There was no intrinsic degeneration of the PIP joint on X-ray or MRI. The right ring finger demonstrated a firm and immobile mass extending for the length of the middle phalanx on the ulnar aspect. Ring finger movement was intact. At the distal aspect of his prior amputation, he had a thickened scar. Mild tenderness to flexion and extension of his bilateral fourth and fifth digits was present. He had prominent fifth metatarsal heads bilaterally which were tender to palpation. Ankle and subtalar joint exam was normal. Ambulation was unrestricted. Multiple large areas of hypertrophic scarring and keloid formation involving the central chest (Figure 3), arms, and legs as well as some discrete nodules on the upper back were present.

**Figure 4:**
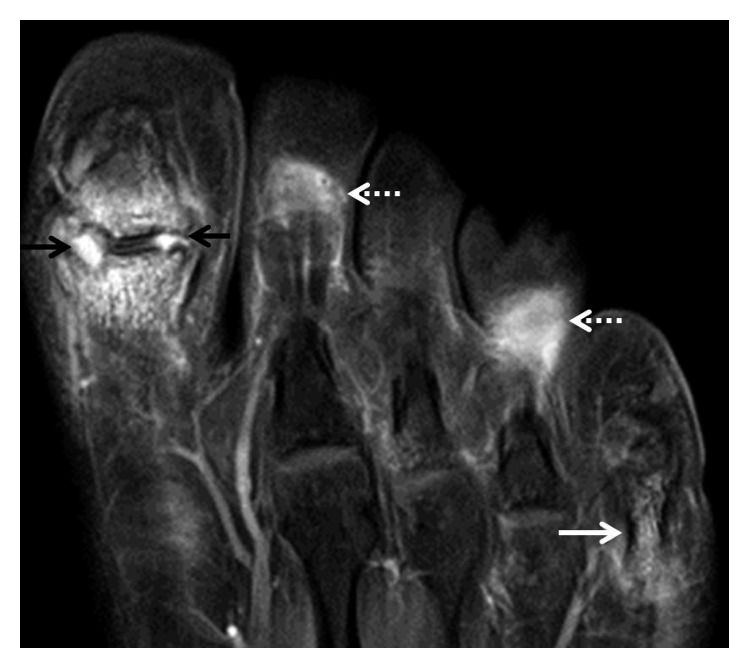
Axial T1 fat-saturated magnetic resonance imaging (MRI) of the foot obtained with gadolinium contrast administration demonstrated marginal erosions at the great toe interphalangeal joint, associated synovitis, and bone marrow enhancement (black arrows). Erosive changes involving the fifth metatarsophalangeal joint were present with bone marrow enhancement in the proximal phalanx (solid white arrow). Enhancing inflammatory changes in the second and fourth toe plantar soft tissues were present (dashed white arrows).

**Figure 5:**
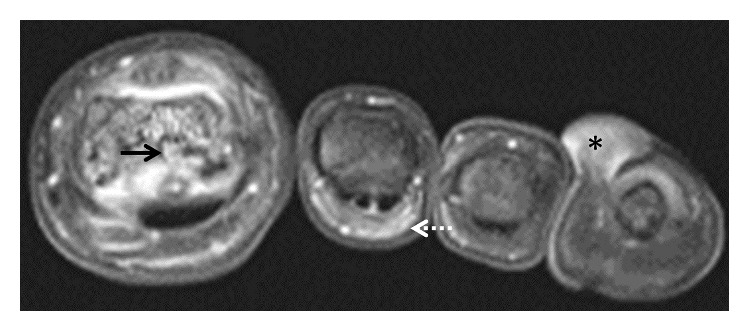
Coronal T1 fat-saturated magnetic resonance imaging (MRI) of the foot obtained with gadolinium contrast administration demonstrated marginal erosions at the great toe interphalangeal joint, associated synovitis, and bone marrow enhancement (black arrows). Enhancing inflammatory changes in the second and fourth toe plantar soft tissues were present (dashed white arrows). One of the several keloids is seen along the medial margin of the fourth toe at the level of the nail with moderate heterogeneous enhancement (asterisk).

Laboratory testing was unremarkable. He tested negative for rheumatoid factor, anticyclic citrullinated peptide, C-reactive protein, erythrocyte sedimentation rate, anti-nuclear antibody, HLA-B27 gene, and hepatitis serology. Routine laboratory markers including complete blood count, renal function, liver function, thyroid and parathyroid gland function, and uric acid and urinalysis were normal. A chest radiograph was unremarkable. A right hand X-ray film showed nonspecific soft tissue swelling of the right ring finger. The left hand film showed amputation of the small finger at the level of the proximal to mid fifth metacarpal and a flexion deformity of the ring finger at the PIP joint. Sacroiliac joints showed minimal degenerative changes on X-rays. Feet radiographs showed multiple erosions. Axial (Figure 4) and coronal (Figure 5) magnetic resonance imaging (MRI) of the feet obtained with gadolinium contrast administration demonstrated marginal erosions, synovitis, and bone marrow enhancement. Enhancing inflammatory changes in the plantar soft tissues and heterogeneous enhancement of keloids was also seen.

To treat the contracture, the patient underwent a left ring finger palmar fasciectomy. Operative findings included severe fibromatosis of the left middle and ring finger and keloid formation that was confirmed by histopathology. After excision of diseased palmar fascia, the finger was able to be fully extended. A residual skin deficit required skin grafting for closure. The entire palmar surface of the proximal phalanx was resurfaced with a full thickness skin graft. His nonoperative skin lesions were treated with a combination of intralesional methylprednisolone, 5-fluorouracil, and pulsed dye laser treatments. He was initiated on 15 mg of oral methotrexate for his erosive disease.

Aggressive occupational therapy was pursued after the surgery. At six-month follow-up, he reported improvement in his joint pain as well as keloids. The ring finger PIP joint flexion contracture had improved from 80° to 35°. However, continued keloid formation persisted over the surgical scars. His skin graft healed completely. Due to the scarring and keloids, no further surgery was recommended.

## 3. Discussion

Palmar fibromatosis is relatively uncommon as compared with keloids. It tends to predominantly occur in males after the age of 40. It has been associated with diabetes, alcohol use, smoking, liver disease, and those using vibratory hand tools [1, 3]. Keloid formation is more common, tends to favor the young, has no gender preference, and spares the hands and the feet. Both diseases can be familial [1, 3].

Polyfibromatosis with arthritis is extremely rare and presents with varying degrees of fibrotic features. Cases of fibromatosis with keloids without arthritic features have also been reported [2, 3]. It is clear that this syndrome presents with variable features as no reported patient has had all syndromic manifestations.  Table 1 summarizes the reported cases of polyfibromatosis with arthritis. Incidence is primarily sporadic. One case was related to phenytoin exposure [4]. Keloid or fibromatosis was the primary reason for clinical presentation. No case presented to a rheumatology department. This likely implies that the arthritic process is secondary, being driven by the fibrotic disease process or that cosmetic concerns are paramount. It is not known if routine radiographic screening of patients with polyfibromatosis would reveal more silent erosive disease than is currently reported. Long-term data are not available on all of these reported patients, but the erosive findings remained unchanged in one patient at 10-year follow-up [10]. In the reported cases, there was no evidence of a systemic rheumatic disease process. Our case had no features of multicentric reticulohistiocytosis or fibroblastic rheumatism. It is interesting to note the presence of synovitis and enhancement of keloids on MRI in our case. A long-term follow-up in our or similar cases may shed more light on the natural history.

The pathogenesis of polyfibromatosis is unclear. Both palmar fibromatosis and keloids have increased production of type III collagen, and anoxia is thought to play an important role. The ischemia produces free oxygen radicals which may promote fibroblastic growth [11]. However, it is thought that the fibroblasts are inherently unstable in keloids in contrast to palmar fibromatosis [10]. One study found increased incidence of carotid atherosclerotic plaque in keloid patients when compared to those with palmar fibromatosis [12]. They speculated that the myofibroblasts in these diseases originated from different cellular precursors. Electron microscopy studies reveal that myofibroblasts are better organized in palmar fibromatosis as opposed to keloids [13]. The etiologic relationship between the musculoskeletal features and polyfibromatosis is unclear. A common triggering factor may be responsible [9]. Bone biopsy done in one case showed increased osteoclast activity [7]. Another common denominator in these conditions may be the matrix metalloproteinases [14–16]. Osteoclast and matrix metalloproteinases could be future therapeutic targets.

Immunosuppressive treatment is generally ineffective although one case responded to methotrexate [6]. Our patient has also had subjective improvement with methotrexate, but only a longer follow-up will reveal its success at prevention of new erosions or halting progression of the current ones. It is unknown if biological agents like tumor necrosis actor inhibitors (TNFi) are useful in this syndrome, but the presence of synovitis in our case and one another [6] indicates its potential usefulness. However, none of the cases had prominent inflammatory joint symptoms despite the aggressive radiological findings. Laboratory studies have shown the inhibitory effects of TNFi in Dupuytren's contracture by inhibiting myofibroblast activity and the contractile apparatus [17]. A phase II clinical trial will assess the role of intralesional adalimumab in early Dupuytren's contracture [18]. Thus, TNFi could possibly help address both the musculoskeletal and dermatological manifestations. Erosions could also possibly be repaired using an osteoclast development inhibitor like denosumab [19] but is untested in this dermatoarthropathy. Bisphosphonates have been used to treat musculoskeletal manifestation in some osteolytic disorders [20].

In conclusion, polyfibromatosis with arthritis is extremely rare with no established treatment. Our case will add to the limited repertoire of erosive polyfibromatosis cases with keloids and serve to increase awareness and thereby stimulate research and reporting of this rare syndrome.

## Figures and Tables

**Table 1:** Summary of polyfibromatosis cases with a musculoskeletal component.

Author/year/country	Age (years)/gender	Fibromatosis	Keloids	Musculoskeletal features	X-ray features	MRI features
Pierard and Lapiere 1979 [4], Belgium	20, male	Hand fibromatosis, knuckle pads, and “toughness” of skin	NA	Camptodactyly, tendon calcification, facial hyoplasia, osteolysis, and scoliosis	Osteolysis of distal ulna, right ring finger proximal phalanx, and first metacarpal	NA

Fenton et al. 1986 [7], United Kingdom	48, male	Palmar fibromatosis, and Peyronie's disease	Keloids of arms, shoulders, and presternal area	Hand and foot stiffness	Erosions of shoulders, hands, and feet	NA

Chen et al. 2006 [5], Australia	65, male	Progressive fibrosis and contractures of wrist, elbows, knees, and ankles; linear fibrotic cords	None	Inflammatory pain of wrist, elbows, knees, and ankles	Erosions of left shoulder, elbows, wrists, hands, and feet	NA

Kim et al. 2009 [8], South Korea	44, male	Palmar and plantar nodules and fibromatosis	Multiple keloids of trunk and extremities	Asymptomatic	Erosions and osteolysis of hands	Multiple hand erosions

Cinotti et al. 2013 [9], Italy	53, male	Flexion contractures of wrists, fingers, ankles, and toes; gingival hyperplasia and conjunctival fibrosis	Multiple keloids	Severe hand and foot deformities and facial changes	Osteolysis of wrists, hands, and toes; ankylosis	NA

Albarran et al. 2015 [6], Spain	33, male	Palmar and plantar nodules, palmar fibromatosis, knee contractures and fibromatosis, and gingival hyperplasia	None	NA	Erosion in right foot	Synovitis and effusions in both knees

Present case 2018, USA	23, male	Finger flexion contractures	Multiple keloids of trunk and extremities	Mild foot pain	Erosions of feet; hands without erosions	Multiple erosions and synovitis of feet
